# Revealing the hidden impact of SGLT2 inhibitors on uric acid levels: a retrospective multicenter cohort study

**DOI:** 10.3389/fendo.2025.1667438

**Published:** 2025-10-20

**Authors:** Ehsan A. Habeeb, Abdullah M. Ghaith, Abdulmajeed M. Alshehri, Ghalia Aquil, Arwa H. Afana, Ali H. Alqarafi, Abdullah A. Alahmed, Omar S. Alkhezi

**Affiliations:** ^1^ Department of Pharmacy Practice, College of Pharmacy, Taibah University, Madinah, Saudi Arabia; ^2^ Prince Mohammad Bin Abdulaziz Hospital, National Guard Health Affairs, Madinah, Saudi Arabia; ^3^ King Abdullah International Medical Research Center, Riyadh, Saudi Arabia; ^4^ Department of Pharmacy Practice, College of Pharmacy, King Saud bin Abdulaziz University for Health Sciences, Riyadh, Saudi Arabia; ^5^ King Abdulaziz Medical City, National Guard Health Affairs, Riyadh, Saudi Arabia; ^6^ Pharmaceutical Care Department, King Faisal Specialist Hospital and Research Center, Jeddah, Saudi Arabia; ^7^ Department of Pharmacy Practice, College of Pharmacy, Qassim University, Qassim, Saudi Arabia

**Keywords:** hyperuricemia, SGLT2 inhibitors, uric acid, diabetes, heart failure, chronic kidney disease

## Abstract

**Introduction:**

Hyperuricemia, characterized by elevated serum uric acid (UA) levels, is associated with cardiovascular–kidney–metabolic syndrome and remains challenging to manage due to medication side effects and adherence issues. SGLT2 inhibitors (SGLT2i), primarily prescribed for diabetes (DM), heart failure (HF), and chronic kidney disease (CKD), have demonstrated potential UA-lowering effects, though their precise impact is not well established.

**Methods:**

This multicenter retrospective cohort study used a pre-and-post analysis to evaluate the effect of SGLT2i on UA levels. Data were collected from four major healthcare centers in Saudi Arabia. The study included adult patients who initiated SGLT2i therapy between January 2022 and January 2024, excluding those with active gout flares, a history of cancer, or recent changes in UA-lowering therapy. The primary outcome was the percentage change in serum UA levels post- i initiation, with secondary outcomes including subgroup analyses, metabolic effects, univariate and multivariate modeling, and longitudinal trend evaluations.

**Results:**

Among 2,400 patients screened, 454 were included in the final analysis. SGLT2i significantly reduced UA levels by 4.5% (p=0.006), with the most pronounced reduction in patients with baseline elevated UA (10%, p=0.001) and those with HF (9%, p=0.001). Univariate analysis identified DM & HF, DM & CKD, and DM, HF & CKD as predictors of response, but multivariate analysis confirmed only DM & HF as an independent predictor (OR = 2.2, 95% CI: 1.2–4.04).

**Conclusion:**

These findings suggest that SGLT2i may serve as an adjunct therapy for hyperuricemia, especially in patients with DM & HF, highlighting the need for further research on long-term benefits.

## 1 Introduction

Hyperuricemia is defined as an elevated serum uric acid (UA) level, typically exceeding 357 μmol/L in women and 416 μmol/L in men (1). Its prevalence varies widely worldwide, ranging from 2.6% to 36%, with a reported rate of 8.4% in Saudi Arabia (2–4). This condition arises from either increased UA synthesis or impaired excretion, ultimately leading to deposition of urate crystals in joints (gout) and kidneys (nephrolithiasis) (5, 6).

Hyperuricemia presents along a clinical spectrum, ranging from asymptomatic elevation of serum UA to manifestations such as gout and nephrolithiasis (7, 8). Importantly, elevated serum UA is independently associated with an increased risk of cardiovascular morbidity and mortality, even in the absence of gout (8–10). Non-pharmacologic interventions play a critical role in managing asymptomatic hyperuricemia, focusing on dietary and lifestyle modifications (11, 12). For symptomatic cases, xanthine oxidase inhibitors remain the first-line pharmacological treatment (13). However, the effectiveness of current urate-lowering therapies is often limited by dosing challenges, adverse effects, and patient nonadherence (14). This underscores the need for exploring alternative therapies that are both safer and more effective.

Sodium-glucose cotransporter-2 inhibitors (SGLT2i), recommended for patients with type 2 diabetes (T2D), heart failure (HF), or chronic kidney disease (CKD), have been shown to reduce serum uric acid (UA) levels through enhanced urinary urate excretion and modulation of metabolic pathways, potentially lowering the risk of incident gout (15–17). However, the magnitude and clinical relevance of this effect, particularly in patients with cardiovascular–kidney–metabolic syndrome, remain uncertain. Although mechanistic and clinical studies suggest that SGLT2i may lower UA through pathways distinct from conventional urate-lowering therapies, findings have not been consistently replicated across diverse populations or real-world settings. Moreover, while large, randomized trials have primarily focused on cardiovascular and renal outcomes, data on the impact of SGLT2i on UA levels in routine clinical practice—especially among patients with multiple comorbidities—remain limited (10, 16). Thus, robust, population-based evidence is needed to clarify the role of SGLT2i in managing hyperuricemia.

Despite advances in hyperuricemia management and a deeper understanding of its relationship with cardiovascular and renal comorbidities, data remain limited regarding the practical effects of SGLT2i on UA levels in real-world patient populations. Observational studies and clinical trials have hinted at the potential of SGLT2i to reduce hyperuricemia through mechanisms distinct from traditional urate-lowering therapies, yet these findings have not been consistently replicated in diverse healthcare settings or in patients with multiple comorbidities such as diabetes, HF, and CKD. Furthermore, while randomized trials have typically focused on cardiovascular and renal endpoints, the impact of SGLT2i on UA levels in routine clinical practice is not fully established, underscoring the need for robust, population-based data in this area. To address this knowledge gap, this study conducted a comprehensive multicenter retrospective cohort utilizing electronic medical records from diverse healthcare institutions across the Kingdom of Saudi Arabia. The primary aim of this study was to evaluate the effect of SGLT2i on serum UA levels in patients with diabetes, heart failure and chronic kidney diseases in different healthcare settings.

## 2 Methods

### 2.1 Study design

This was a multicenter retrospective cohort study of patients who received an SGLT2i for any indication. Only patients newly initiated on an SGLT2i between January 2022 and January 2024 were eligible; patients already receiving therapy prior to this period were excluded. Data were collected from three tertiary care hospitals and one cardiac center in Saudi Arabia. The study was approved by the Institutional Review Board (NRC24R/081/02) on July 4th, 2024. Eligible patients were aged 18 years or older and received an SGLT2i for DM, HF, or CKD. Exclusion criteria included active gout flares, a history of cancer, or initiation or dose adjustment of urate-lowering therapy within 90 days before SGLT2i initiation or any changes in urate-lowering therapy while on SGLT2i. Baseline uric acid was defined as the most recent measurement within six months before SGLT2i initiation; if multiple values were available, the average was recorded. The included patients had a minimum follow-up duration of 10 months. Elevated serum UA levels were defined as >420 μmol/L (~7.0 mg/dL) in males or >360 μmol/L (~6.0 mg/dL) in females (18). Chronic NSAID use was defined as daily or most-days of the week for at least 3 months, consistent with widely accepted definitions (19).

### 2.2 Study objective and data collection

The primary outcome was the percentage change in serum UA levels following SGLT2i initiation compared to baseline prior of medications initiation. Secondary outcomes included changes in metabolic parameters and adverse effects. Baseline clinical data collected included age, sex, body mass index (BMI), comorbidities, type and dose of SGLT2i. Laboratory parameters included UA (μmol/L), eGFR (mL/min/1.73 m²), left ventricular ejection fraction (LVEF%) and glycated hemoglobin (A1C%). Data on gout medications and anti-inflammatory therapies were also collected.

### 2.3 Statistical analysis

Nominal data were analyzed using Chi-square or Fisher’s exact tests, while continuous data were analyzed using the Mann–Whitney U test, Student t-test, or Wilcoxon signed-rank test, as appropriate, with a significance level of α=0.05. The Shapiro-Wilk test was used to assess normality of continuous data. As UA levels were not normally distributed, the Wilcoxon signed-rank test was used for the primary outcome. For univariate analysis, responders were defined as patients achieving a ≥8.5% reduction in UA levels after SGLT2i initiation. This threshold was selected to ensure clinically significant reductions were captured even among patients with normal or near-normal baseline UA levels. Similar percentage-based thresholds have been utilized in previous studies and guidelines evaluating urate-lowering therapies that highlighted the clinical relevance of percentage reductions when assessing treatment response in gout and hyperuricemia management (20, 21). Multivariate analysis was performed to adjust for potential confounders, including all variables with P ≤ 0.2 in univariate analysis. Longitudinal regression modeling was also conducted to assess trends in UA reductions over time. Longitudinal analyses were based on routine uric acid measurements obtained after SGLT2i initiation, which were organized into 2-month intervals; values within each interval were plotted to assess temporal trends. All statistical analyses were performed using Stata Statistical Software or SPSS Statistics (licensed by the affiliated universities).

## 3 Results

### 3.1 Patient population

**Figure 1:**
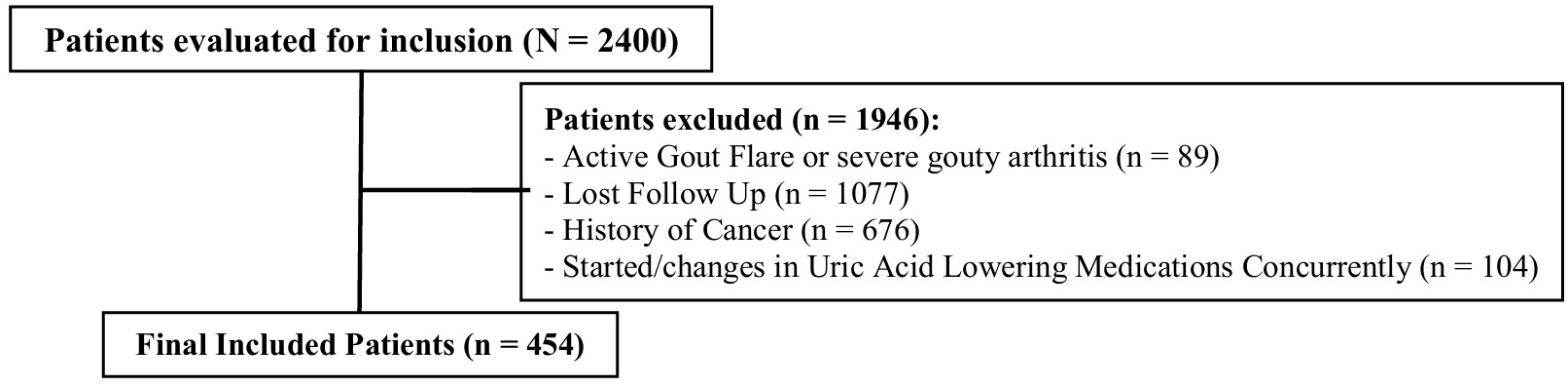
Flow diagram of patient inclusion in the study.

Data were collected from three tertiary care hospitals and one cardiac center in Saudi Arabia. Of the 2,400 patients screened, 1,946 were excluded, primarily due to loss to follow-up. The second most common exclusion criterion was a history of cancer. Additional details on exclusion criteria are shown in **Figure 1**. Ultimately, 454 patients were included in the final analysis. Most patients (91.8%) initiated SGLT2i in the outpatient setting. The baseline characteristics of the included patients are summarized in **Table 1**. The median age was 63 years, with 62% male and a median BMI of 29.7 kg/m². Empagliflozin was the more frequently used SGLT2i (57%), compared to dapagliflozin (43%), largely driven by formulary differences across sites. All patients received the standard dose of dapagliflozin (10 mg daily) or empagliflozin (10 mg daily), with no dose adjustments during follow-up. A diagnosis of DM was documented in 70% of patients, with most having been diagnosed more than 5 years prior to SGLT2i initiation. Heart failure (HF) and chronic kidney disease (CKD) were reported in 66.7% and 67% of patients, respectively, with disease durations typically between 1–5 years. Among HF patients, reduced ejection fraction was more common (67.3%) than preserved ejection fraction (32.7%). Only 3 patients initiated SGLT2i therapy exclusively for CKD without other comorbidities, while 23% had the combined comorbidity of DM, HF, and CKD. Elevated UA levels before SGLT2i initiation were observed in 25% of patients.

**Table 1:** Baseline characteristics.

Variables	Included patients (n = 454)
Age (years)^*^	63 ± 10.4
Gender, male^†^	282 (62)
BMI (kg/m^2^)^╝^	29.7 [26:33.7]
Hospital prescribing setting^†^	
SGLT2i Prescribed outpatient or upon discharge	417 (91.8)
SGLT2i Prescribed inpatient	37 (8.2)
SGLT2 inhibitor agent ^†^	
Empagliflozin	259 (57)
Dapagliflozin	195 (43)
Indication of SGLT2 Inhibitors ^†^	(N = 454)
Diabetes Mellitus (DM)	(n= 321) (70)
DM and Heart Failure only	71 (22)
DM and Chronic Kidney Disease (CKD) only	113 (35)
Duration of Having Diabetes, < 1 year	41 (12.7)
Duration of Having Diabetes, 1–5 years	83 (25.9)
Duration of Having Diabetes, > 5 years	197 (61.4)
Heart Failure (HF)	(n= 303) (66.7)
Heart Failure Reduced (HFrEF)	204 (67.3)
Heart Failure and CKD only	88 (29)
Duration of Having HF, < 1 year	56 (18.5)
Duration of Having HF, 1–5 years	202 (67)
Duration of Having HF, > 5 years	45 (14.5)
Chronic Kidney Disease	306 (67)
Duration of Having CKD, < 1 year	24 (8.6)
Duration of Having CKD, 1–5 years	186 (60)
Duration of Having CKD, > 5 years	96 (31.4)
All Combined Comorbidity (DM, HF and CKD)	102 (22.5)
Patients with elevated UA level before starting SGLT2 Inhibitor ^†^	111 (24.5)
Duration of elevated UA level, < 1 year	13 (11.7)
Duration of elevated UA level, 1–5 year	93 (84)
Duration of elevated UA level, > 5 years	5 (4.3)

* Mean ± standard deviation; ╝: Median [interquartile range]; † n (%); BMI, Body-Mass Index; SGLT2, Sodium Glucose Cotransporter 2; UA, Uric Acid.

### 3.2 Outcomes

**Table 2** representing the primary endpoint, shows a significant reduction in serum UA levels following the initiation of SGLT2i. For the total patient cohort, the median UA level decreased from 336 [315–368] μmol/L to 321 [295–350] μmol/L, reflecting a 4.5% reduction (p=0.006). The most pronounced decrease was observed in patients with baseline elevated UA levels, with a 10% reduction (from 425 [371–467] to 383 [340–433] μmol/L, p=0.001). Subgroup analysis demonstrated statistically significant reductions in UA levels across various patient groups. Males experienced a 4.5% decrease (p=0.006), while females had a 3.2% reduction (p=0.01). Patients with HF exhibited a 9% reduction (p=0.001), with median levels decreasing from 349 [327–399] to 318 [286–366] μmol/L. Reductions were also notable in patients with both HF and DM. Additional subgroup analyses are detailed in **Table 2**.

**Table 2:** Primary endpoint.

Primary endpoint ^╝^	UA level before SGLT2I	UA level after starting SGLT2I	Percentage of decrease	P value
Total Patients	336 [315 - 368]	321 [295 - 350]	4.5%	**0.006**
Subgroups Per Primary Endpoint ^╝^				
Patient with Baseline Elevated UA Levels (n= 111)	425 [371 - 467]	383 [340 - 433]	10%	**0.001**
Male (n= 282)	335 [314 - 367]	320 [287 - 344]	4.5%	**0.006**
Female (n= 172)	340 [317 - 370]	329 [302 - 367]	3.2%	**0.01**
Patients with DM only (n= 35)	333 [308 – 350]	316 [298 - 348]	5%	**0.006**
Patients with HF only (n= 42)	349 [327 – 399]	318 [286 - 366]	9%	**0.001**
Patients with DM and HF (n= 71)	337 [314 – 365]	312 [263 - 355]	7%	**0.005**
Patients with DM and CKD (n=113)	333 [315 – 358]	321 [304 - 347]	3.6%	**0.01**
Patients with HF and CKD (n= 88)	338 [314 – 388]	322 [302 - 385]	5%	**0.006**
Patients with DM, HF and CKD (n= 102)	334 [315 – 366]	321 [297 - 343]	3.8%	**0.01**
Patients on UA-Lowering Home Medications (n= 33)	329 [304 – 379]	318 [288 – 352]	3.3%	**0.01**

╝: Median [interquartile range]; UA, Uric Acid; SGLT2, Sodium Glucose Cotransporter 2; DM; Diabetes Mellitus; HF, Heart Failure; CKD, Chronic Kidney Disease; Statistical test used, Wilcoxon signed-rank test. Bold values indicate statistically significant results (p < 0.05).

**Figure 2:**
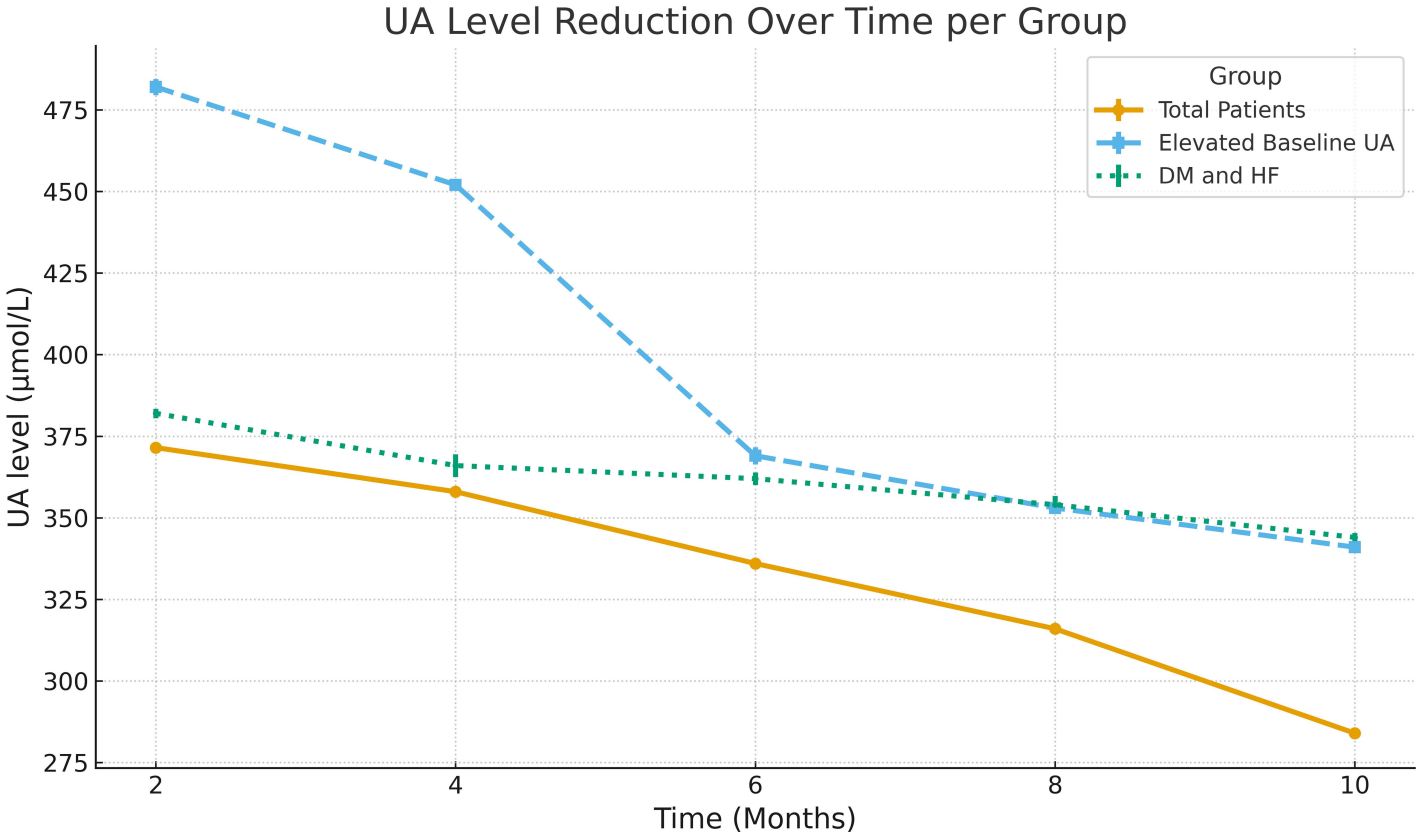
Longitudinal regression showing the reduction in serum uric acid (UA) levels over time across patient subgroups.

Univariate analysis revealed that patients with baseline elevated UA level (p=0.001), those with DM & HF (p=0.001), DM & CKD (p=0.02), and DM, HF, & CKD (p=0.002) were significantly more likely to be responders, while other variables, including age, sex, and SGLT2i type, did not show significant associations. In multivariate analysis, baseline elevated UA level (OR = 2.6, 95% CI: 1.9-5.1, p=0.002) and DM & HF (OR = 2.2, 95% CI: 1.2–4.04, p=0.007) remained an independent predictor of response. Associations for DM & CKD (OR = 0.73, p=0.26), DM, HF, & CKD (OR = 0.52, p=0.12), HF-only patients (OR = 1.7, p=0.17) and male sex (OR = 1.4, p=0.11) did not demonstrate significant independent effects (**Table 3**). **Figure 2** illustrates UA level reductions over time for three patient groups. The line for “Total Patients” shows a consistent, gradual decline over 10 months. The line for “Elevated Baseline UA” shows a more rapid decrease, especially around months 5 to 6. The line for “DM and HF” shows a steep initial decline around month 3, then a stable pattern through month 10.

**Table 3:** Univariate and multivariate analysis.

Variable	Univariate analysis			Multivariate analysis	
	Responders (n = 168)	Non-responders (n = 286)	P-value	Odds ratio (95% CI)	P-value
Age, years *	63 ± 10	63 ± 10.5	0.92	–	–
Male ^†^	111 (66.1)	171 (60)	0.19	1.4 [0.93 – 2.1]	0.11
Female^†^	57 (34)	115 (40)	0.18	–	–
Patients with baseline elevated UA levels^†^	59 (36)	52 (18)	**0.001**	**2.6 [1.9 – 5.1]**	**0.002**
SGLT2 Inhibitor ^†^					
Empagliflozin	102 (61)	157 (55)	0.22	–	
Dapagliflozin	66 (39.3)	129 (45)	0.22	–	
Indication^†^					
Patients with DM only	12 (7)	23 (8)	0.73	–	–
Patients with HF only	21 (12.5)	21 (7.3)	0.06	1.7 [0.8 – 3.4]	0.17
Patients with DM & HF	39 (23)	32 (11)	**0.001**	**2.2 [1.2 – 4.04]**	**0.007**
Patients with DM & CKD	32 (19)	81 (28)	0.02	0.73 [0.42 – 1.3]	0.26
Patients with HF & CKD	30 (18)	58 (20)	0.52	–	–
DM, HF and CKD	54 (32)	48 (17)	**0.002**	0.52 [0.22 – 1.2]	0.12

* Mean ± standard deviation; ╝: Median [interquartile range]; † n (%); SGLT2, Sodium Glucose Cotransporter 2; DM, diabetes mellitus; HF, heart failure; CKD, chronic kidney disease. Bold values indicate statistically significant results (p < 0.05).

**Table 4** presents the secondary endpoints evaluated in this study. There were no significant changes in eGFR 66 [50 - 77] vs 67 [53 - 78] (p=0.73) or LVEF (p=0.98) following SGLT2i initiation. However, there was a significant reduction in HbA1c levels from 8.1 ± 1.9% to 7.3 ± 1.5% (p=0.001). Adverse events were rare, with four cases (0.8%) of ketoacidosis and 38 cases (8.4%) of urinary tract infections. No cases of necrotizing fasciitis or limb amputation were observed. Regarding home medications, 33 patients were receiving agents that may influence uric acid, including NSAIDs (n=14, 3.1%), systemic steroids (n=11, 2.4%), colchicine (n=2, 0.4%), and allopurinol (n=6, 1.3%). No changes in dosing were observed during the study period based on chart review and refill history.

**Table 4:** Secondary endpoints.

Variables	Before SGLT2 inhibitors	After starting SGLT2 inhibitors	*P*-value
eGFR ^╝^	66 [50 - 77]	67 [53 – 78]	0.73
LVEF *	46 ± 9.4	47 ± 9.3	0.98
HA1C *	8.1 ± 1.9	7.3 ± 1.5	0.001
Other Secondary Endpoints†:			
Incidence of Ketoacidosis	4 (0.8)		
Incidence of Urinary Tract Infection	38 (8.4)		
Incidence of Necrotizing Fasciitis	0 (0)		
Incidence of Limp Amputation	0 (0)		
Home Medications:†			
Regular Use of NSAID	14 (3.1)		
On Systematic Steroid	11 (2.4)		
On Colchicine	2 (0.4)		
On Allopurinol	6 (1.3)		

* Mean ± standard deviation; † n (%); eGFR, estimated Glomerular Filtration Rate; LVEF, Left Ventricular Ejection Fraction; HA1C, Hemoglobin A1C; SGLT2, Sodium Glucose Cotransporter 2; DM, diabetes mellitus; HF, heart failure; CKD, chronic kidney disease.

## 4 Discussion

This study examines the effect of SGLT2i on UA levels in a real-world, multicenter cohort in Saudi Arabia. Conducted across four major medical centers, this analysis demonstrated that SGLT2i therapy reduced UA levels regardless of the indication for use. Although patients with elevated baseline UA experienced the most pronounced reductions, UA-lowering effects were consistent across multiple subgroups. Additionally, the presence of both DM and HF emerged as an independent predictor of a favorable UA response. Given the high prevalence of hyperuricemia in patients with DM, HF, and CKD, - and its known association with worse outcomes - these findings support the use of SGLT2i in these patient populations (22–24).

In this cohort, about 35% of patients with DM also had CKD, aligning with previous findings in the Saudi population (25). Similarly, approximately 25% of patients with DM in this study also had HF, reflecting the well-documented bidirectional relationship between these conditions, where diabetes can contribute to structural and functional changes in the heart, and HF can exacerbate metabolic abnormalities, further increasing diabetes risk (26). For patients with combined diabetes, HF, and CKD, the prevalence was estimated at around 15–20% in this cohort, consistent with the concept of the “cardiorenal-metabolic syndrome,” where these interconnected conditions significantly worsen outcomes due to shared pathophysiological mechanisms (27). Additionally, among patients with HF, CKD prevalence in this study was 23%, which is within the reported range in previous epidemiological studies (28).

In this study, SGLT2i treatment led to a 4.5% reduction in UA levels over a 10-month follow-up. While this reduction is modest, it aligns with previous *post hoc* analyses of randomized clinical trials that reported approximately 13% reduction in UA levels with canagliflozin compared to placebo in type 2 DM patients (29). Similarly, empagliflozin lowered UA levels in the EMPA-REG OUTCOME study (30). A recent meta-analysis of patients with and without type 2 diabetes (n ≈ 464,009) showed that SGLT2i reduce the risk of clinically relevant hyperuricemic events by 33% (HR, 0.67; 95% CI, 0.59–0.77), reinforcing the UA-lowering effects beyond diabetic populations (31). Real-world studies have also confirmed these effects, and empagliflozin has demonstrated UA-lowering benefits in HF patients regardless of ejection fraction phenotype (24, 29).

Although this study did not assess gout incidence directly, prior analyses of trials such as EMPA-REG OUTCOME, EMPEROR-Reduced, CANVAS, and EMPEROR-Preserved—as well as real-world data—have shown that SGLT2i use reduces gout incidence (24, 32–34). Notably, SGLT2i have also been associated with lower risk of recurrent gout flares compared to GLP-1 receptor agonists and DPP-4 inhibitors in type 2 DM patients (35).

The UA-lowering effects of SGLT2i likely extend beyond their uricosuric action, involving reductions in UA synthesis via downregulation of pentose phosphate pathway enzymes (17, 36). Although some studies suggest that urate-lowering therapies like allopurinol can reduce cardiovascular events in hypertension, this effect has not consistently translated to HF populations (37, 38). Thus, the UA-lowering properties of SGLT2i may contribute to—but not solely account for—their cardiovascular benefits.

It is noteworthy that most patients with DM in this cohort initiated SGLT2i approximately 5 years after diagnosis. Earlier initiation may help prevent complications such as CKD, highlighting the importance of timely adherence to current guidelines (15, 39, 40). Given the high prevalence and adverse outcomes of hyperuricemia in patients with DM, HF, and CKD, the UA-lowering effect of SGLT2i may represent an additional pleiotropic benefit in these populations. While this study did not assess gout incidence or flare reduction, future prospective studies are warranted to determine whether these metabolic effects translate into improved gout-related outcomes (15, 39).

This study’s real-world, multicenter design across four major healthcare centers in Saudi Arabia enhances generalizability. The main limitation is the absence of a comparator group. As SGLT2i are now guideline-recommended for patients with diabetes, heart failure, and chronic kidney disease, an untreated control group was not feasible retrospectively. Instead, a pre–post design was used, allowing patients to serve as their own controls and reducing inter-individual variability. While this does not replace a formal comparator, the findings remain consistent with prior randomized trials. Another limitation is the lack of detailed data on diet and concomitant medications (e.g., diuretics), although we excluded patients with recent changes in urate-lowering therapy to minimize confounding. Moreover, the high exclusion rate, largely reflecting patients lost to follow-up. These exclusions were necessary to preserve data quality and ensure valid assessment of uric acid changes, but they may still introduce selection bias and affect generalizability.

## 5 Conclusion

SGLT2i were associated with a modest but consistent reduction in uric acid, most pronounced in patients with elevated baseline levels and those with both diabetes and heart failure. These findings suggest potential metabolic benefits and should be regarded as hypothesis-generating, warranting further prospective studies to confirm and clarify their implications for long-term patient management.

## Data Availability

The original contributions presented in the study are included in the article/supplementary material. Further inquiries can be directed to the corresponding author.
